# Correction: Service availability and readiness of malaria surveillance information systems implementation at primary health centers in Indonesia

**DOI:** 10.1371/journal.pone.0292827

**Published:** 2023-10-09

**Authors:** Maria Holly Herawati, Besral, Dina Bisara Lolong, Noer Endah Pracoyo, Noor Edi Widya Sukoco, Hadi Supratikta, Meita Veruswati, Al Asyary

There are errors in the Funding section. The correct Funding statement is: This publication of this study is funded by the Universitas Indonesia PUTI 2023 Award: NKB-432/UN2.RST/HKP.05.00/2023. This research was also supported by the National Institute for Research and Development, Indonesian Ministry of Health which has been integrated into Indonesian National Research and Innovation Agency (BRIN). The funders had no role in study design, data collection and analysis, decision to publish, or preparation of the manuscript.
