# Supplementary material for: Service availability and readiness of malaria surveillance information systems implementation at primary health centers in Indonesia
Source: PLoS One. 2023 Apr 27;18(4):e0284162. doi: 10.1371/journal.pone.0284162 (PMC10138467; doi:10.1371/journal.pone.0284162)
Supplement: Attachment — Submitted filename: PONE-D-22-07674_reviewer_comments_CO.pdf [file pone.0284162.s001.pdf]

PLOS ONE

Service Availability and Readiness of Malaria Surveillance Information Systems Implementation at Primary Health Centers in Indonesia

--Manuscript Draft--

**Manuscript Number:**	PONE-D-22-07674
**Article Type:**	Research Article
**Full Title:**	Service Availability and Readiness of Malaria Surveillance Information Systems Implementation at Primary Health Centers in Indonesia
**Short Title:**	Malaria Surveillance Information System in Indonesia
**Corresponding Author:**	Meita Veruswati<br>Universitas Muhammadiyah Prof Dr Hamka<br>INDONESIA
**Keywords:**	malaria; information system; availability; readiness; Indonesia
**Abstract:**	One of the most important indicators in malaria eradication is the malaria surveillance information system (SISMAL) for recording and reporting medical cases. This paper aims to describe the availability and readiness of SISMALs at primary health centers (PHCs) in Indonesia. A cross-sectional survey was implemented in seven provinces for this study. The data was analyzed using bivariate, multivariate, and linear regression. The availability of the information system was measured by assessing the presence of the electronic malaria surveillance information system (E-SISMAL) at the studied PHCs. The readiness was measured by averaging each component of the assessment. From 400 PHC samples, only 58.5% had available SISMALs, and their level of readiness was only 50.2%. Three components had very low levels of readiness: (1) the availability of personnel (40.9%), (2) SISMAL integration and storage (50.2%), and (3) the availability of data sources and indicators (56.8%). Remote and border (DTPK) areas had a 4% better readiness score than non-DTPK areas. Endemic areas were 1.4% better than elimination areas, while regions with low financial capacity were 3.78% better than regions with high financial capacity, with moderate capacity (2.91%). The availability rate of the SISMAL at PHCs is only 58.5%. Many PHCs still do not have SISMALs. The readiness of the SISMAL at these PHCs is significantly related to DTPK area, endemicity status, and financial capacity.
**Order of Authors:**	Maria Holly Herawati, PhD
	Besral Besral, PhD
	Al Asyary, PhD
	Meita Veruswati
**Additional Information:**	
**Question**	**Response**
**Financial Disclosure**<br><br>Enter a financial disclosure statement that describes the sources of funding for the work included in this submission. Review the submission guidelines for detailed requirements. View published research articles from *PLOS ONE* for specific examples.<br><br>This statement is required for submission and **will appear in the published article** if the submission is accepted. Please make	This research was funded by NIHRD Ministry of Health Republic of Indonesia.

Powered by Editorial Manager® and ProduXion Manager® from Aries Systems Corporation

sure it is accurate.<br><br>**Unfunded studies**<br>Enter: *The author(s) received no specific funding for this work.*<br><br>**Funded studies**<br>Enter a statement with the following details:<br>• Initials of the authors who received each award<br>• Grant numbers awarded to each author<br>• The full name of each funder<br>• URL of each funder website<br>• Did the sponsors or funders play any role in the study design, data collection and analysis, decision to publish, or preparation of the manuscript?<br>• **NO** - Include this sentence at the end of your statement: *The funders had no role in study design, data collection and analysis, decision to publish, or preparation of the manuscript.*<br>• **YES** - Specify the role(s) played.<br><br>* typeset	
**Competing Interests**<br><br>Use the instructions below to enter a competing interest statement for this submission. On behalf of all authors, disclose any competing interests that could be perceived to bias this work—acknowledging all financial support and any other relevant financial or non-financial competing interests.<br><br>This statement is required for submission and **will appear in the published article** if the submission is accepted. Please make sure it is accurate and that any funding sources listed in your Funding Information later in the submission form are also declared in your Financial Disclosure statement.<br><br>View published research articles from *PLOS ONE* for specific examples.	The authors have declared that no competing interests exist.

**NO authors have competing interests**<br><br>Enter: *The authors have declared that no competing interests exist.*<br><br>**Authors with competing interests**<br><br>Enter competing interest details beginning with this statement:<br><br>*I have read the journal's policy and the authors of this manuscript have the following competing interests: [insert competing interests here]*<br><br>* typeset	
**Ethics Statement**<br><br>Enter an ethics statement for this submission. This statement is required if the study involved:<br><br>• Human participants<br>• Human specimens or tissue<br>• Vertebrate animals or cephalopods<br>• Vertebrate embryos or tissues<br>• Field research<br><br>Write "N/A" if the submission does not require an ethics statement.<br><br>General guidance is provided below. Consult the submission guidelines for detailed instructions. **Make sure that all information entered here is included in the Methods section of the manuscript.**	The study was conducted in accordance with the Declaration of Helsinki, and approved by the Institutional Review Board (or Ethics Committee) of (Protocol Code: Leader of Centre of Research and Development for Human Resources and Health Services, National Institute for Health Re-search and Development, Indonesian Ministry of Health, No. HK.03.02/1/180/2019, and Ethical Approval LB.02.01/2/KE.186/2019).

Powered by Editorial Manager® and ProduXion Manager® from Aries Systems Corporation

**Format for specific study types**<br><br>**Human Subject Research (involving human participants and/or tissue)**<br>• Give the name of the institutional review board or ethics committee that approved the study<br>• Include the approval number and/or a statement indicating approval of this research<br>• Indicate the form of consent obtained (written/oral) or the reason that consent was not obtained (e.g. the data were analyzed anonymously)<br><br>**Animal Research (involving vertebrate animals, embryos or tissues)**<br>• Provide the name of the Institutional Animal Care and Use Committee (IACUC) or other relevant ethics board that reviewed the study protocol, and indicate whether they approved this research or granted a formal waiver of ethical approval<br>• Include an approval number if one was obtained<br>• If the study involved *non-human primates*, add *additional details* about animal welfare and steps taken to ameliorate suffering<br>• If anesthesia, euthanasia, or any kind of animal sacrifice is part of the study, include briefly which substances and/or methods were applied<br><br>**Field Research**<br><br>Include the following details if this study involves the collection of plant, animal, or other materials from a natural setting:<br>• Field permit number<br>• Name of the institution or relevant body that granted permission	
**Data Availability**<br><br>Authors are required to make all data underlying the findings described fully available, without restriction, and from the time of publication. PLOS allows rare exceptions to address legal and ethical concerns. See the PLOS Data Policy and FAQ for detailed information.	Yes - all data are fully available without restriction

A Data Availability Statement describing where the data can be found is required at submission. Your answers to this question constitute the Data Availability Statement and **will be published in the article**, if accepted.<br><br>**Important:** Stating 'data available on request from the author' is not sufficient. If your data are only available upon request, select 'No' for the first question and explain your exceptional situation in the text box.<br><br>Do the authors confirm that all data underlying the findings described in their manuscript are fully available without restriction?	
**Describe where the data may be found in full sentences. If you are copying our sample text, replace any instances of XXX with the appropriate details.**<br><br>• If the data are **held or will be held in a public repository**, include URLs, accession numbers or DOIs. If this information will only be available after acceptance, indicate this by ticking the box below. For example: *All XXX files are available from the XXX database (accession number(s) XXX, XXX.)*.<br>• If the data are all contained **within the manuscript and/or Supporting Information files**, enter the following: *All relevant data are within the manuscript and its Supporting Information files.*<br>• If neither of these applies but you are able to provide **details of access elsewhere**, with or without limitations, please do so. For example:<br><br>*Data cannot be shared publicly because of [XXX]. Data are available from the XXX Institutional Data Access / Ethics Committee (contact via XXX) for researchers who meet the criteria for access to confidential data.*<br><br>*The data underlying the results presented in the study are available from (include the name of the third party*	Data are available from the National Institute for Health Research and Development (NIHRD) Institutional Data Access / Ethics Committee (contact via Dr Maria Holly Herawati) for researchers who meet the criteria for access to confidential data.

and contact information or URL).<br>• This text is appropriate if the data are owned by a third party and authors do not have permission to share the data.<br><br>* typeset	
Additional data availability information:	Tick here if the URLs/accession numbers/DOIs will be available only after acceptance of the manuscript for publication so that we can ensure their inclusion before publication.; Tick here if your circumstances are not covered by the questions above and you need the journal's help to make your data available.

Powered by Editorial Manager® and ProduXion Manager® from Aries Systems Corporation

Click here to access/download;Manuscript;Malaria_MH HerawatiPONE.docx

Article

Service Availability and Readiness of Malaria Surveillance Information Systems Implementation at Primary Health Centers in Indonesia

Maria Holly Herawati [1], Besral [2], Al Asyary [3] and Meita Veruswati [4,*]

[1] Centre of Research and Development for Human Resources and Health Services, National Institute for Health Research and Development, Indonesian Ministry of Health; mariahollyherawati@gmail.com
[2] Department of Biostatistics and Population Studies, Faculty of Public Health, Universitas Indonesia; besral@ui.ac.id
[3] Department of Environmental Health, Faculty of Public Health, Universitas Indonesia; alasyary@ui.ac.id
[4] Study Program of Public Health, Faculty of Health Sciences, Universitas Muhammadiyah Prof. Dr. HAMKA, Jakarta, Indonesia; meitaveruswati@uhamka.ac.id
* Correspondence: meitaveruswati@uhamka.ac.id; Tel.: (optional; include country code; if there are multiple corresponding authors, add author initials)

Abstract: One of the most important indicators in malaria eradication is the malaria surveillance information system (SISMAL) for recording and reporting medical cases. This paper aims to describe the availability and readiness of SISMALs at primary health centers (PHCs) in Indonesia. A cross-sectional survey was implemented in seven provinces for this study. The data was analyzed using bivariate, multivariate, and linear regression. The availability of the information system was measured by assessing the presence of the electronic malaria surveillance information system (E-SISMAL) at the studied PHCs. The readiness was measured by averaging each component of the assessment. From 400 PHC samples, only 58.5% had available SISMALs, and their level of readiness was only 50.2%. Three components had very low levels of readiness: (1) the availability of personnel (40.9%), (2) SISMAL integration and storage (50.2%), and (3) the availability of data sources and indicators (56.8%). Remote and border (DTPK) areas had a 4% better readiness score than non-DTPK areas. Endemic areas were 1.4% better than elimination areas, while regions with low financial capacity were 3.78% better than regions with high financial capacity, with moderate capacity (2.91%). The availability rate of the SISMAL at PHCs is only 58.5%. Many PHCs still do not have SISMALs. The readiness of the SISMAL at these PHCs is significantly related to DTPK area, endemicity status, and financial capacity.

Keywords: malaria; information system; availability; readiness; Indonesia , Surveillance

1. Introduction

As a tropical country, Indonesia is endemic to tropical diseases such as malaria.[1, 2] The malaria control program has been in effect since 1914, during the Dutch colonial period.[3] Indonesia National Health Day was first held on November 12, 1959, when President Sukarno symbolically sprayed pesticide against malaria mosquitoes in the Kalasan village, a special region of Yogyakarta Province.[4] The 2019 malaria control report states that out of 514 districts/cities in Indonesia, 300 districts were declared ~~eliminated (~~free of malaria~~)~~ (58%), 160 were slightly endemic (31%), 31 were moderately endemic (6%), and 23 were highly endemic (5%).[5]

The malaria control program in Indonesia aims, by 2030, to achieve total malaria elimination (malaria free) in stages. [illegible] steps taken are to grant a malaria certification per region, starting from the district/city to the provincial, regional, and national levels. The

term "regional certification" arises because the area of Indonesia is huge, and thus, the capacities of health services, population, and regional infrastructure are varied. There are five areas of malaria regionalization, covering the Java and Bali regions, Sumatra, the West Nusa Tenggara and Sulawesi regions, the Kalimantan and North Maluku regions, the NTT and Maluku regions, and the West Papua and Papua regions.[5]

The malaria control program was developed to expand the public health effort. Good-quality health development requires system strengthening. According to the World Health Organization (WHO), an aspect of such strengthening is the information system.[6] This will increase the validity and completeness of malaria data reporting. According to Law No. 4 of 1984, malaria is an endemic infectious disease with the potential to be an epidemic. Therefore, epidemiological recording or surveillance reports must be supported on an ongoing basis.[7, 8] The legal basis for strengthening the information system of malaria data reporting is stated in the decree of the minister of health of the Republic of Indonesia (Number 293/2009) regarding the elimination of malaria in Indonesia. It was stated in the sixth strategy, i.e., to organize a surveillance system, with monitoring and evaluation, as well as a health information system for malaria. Moreover, the third pillar of the Global Technical Strategy for Malaria 2016–2030, the transformation of malaria surveillance, is a core indicator of the importance of strengthening such a system.[9, 10]

The use of information systems in the malaria control program is essential in the capacity building of the program.[9, 11] Information systems guarantee the accuracy of data collection and analysis to support decision making. The information system governance approach is a strategic effort used as the basis for policies at various levels of health services and at all levels of government in response to the challenges of the times. In 2012, an electronic-based application of the malaria surveillance information system (SISMAL) was developed for the recording and reporting of malaria control. This application has been implemented in several districts. Then in 2015, the electronic malaria surveillance information system (E-SISMAL) was implemented in health services. The E-SISMAL software was established to improve validity and completeness in reporting malaria data.[10] One of the requirements for a district/city to obtain a malaria elimination certificate is a complete malaria case register, which includes a complete elimination area.[11, 12, 13]

Since 2018, the second version of the E-SISMAL has been disseminated to all provinces. This version utilizes a web-based system to improve performance and data quality.[10]

This article aims to assess and discuss the availability and readiness of the information system for malaria control programs in health-care centers, i.e., the E-SISMAL. The data was analyzed from National Health Facility Research (Rifaskes) in combination with data collected from a thematic study of health information systems in 2019.[14]

2. Materials and Methods

Data was collected from seven purposively selected provinces, with the specific consideration of certain factors, i.e., the regional representativeness of BPJS Kesehatan (the Health Care and Social Security Agency) and different territories of Indonesia. These provinces are Aceh, West Java, West Nusa Tenggara, West Kalimantan, Central Sulawesi, South Sulawesi, and Papua. From the 7 provinces, we randomly selected 103 districts/cities and 400 health centers. We interviewed the head and staff of the Malaria program at each health-care center.[14] We then recruited a local interviewer who had been involved with the Rifaskes in 2019. Training sessions for the interviewer were held virtually and during face-to-face meetings. Direct supervision by the research team was carried out during data collection.[14]

The data was analyzed using bivariate, multivariate, and linear regression analysis. The availability of the information system was measured by assessing the presence or absence of the E-SISMAL at primary health centers (PHCs). Data analysis was assessed based on province, remote area, regional financial capacity, and regional status according to the malaria program, and each area was classified as either eliminated or endemic (low,

medium, high, or combined). The readiness of the SISMAL was measured by averaging each component of the assessment, which includes organization, availability of personnel, availability of infrastructure, data sources and indicators, monitoring and evaluation data and verification, data quality, presentation, utilization and feedback, data integration, and storage data.

The procedures followed were in accordance with the ethical standards of the committee on human experimentation (institutional and national) (Protocol Code: Leader of Centre of Research and Development for Human Resources and Health Services, National Institute for Health Research and Development, Indonesian Ministry of Health, No. HK.03.02/1/180/2019, and Ethical Approval LB.02.01/2/KE.186/2019).[14]

3. Results

3.1. Availability of Malaria Surveillance Information Systems at Primary Health Centers

Of all the 400 PHCs in 7 provinces, only 58.5% had malaria program information systems available. The three provinces with the lowest availability of SISMALs at PHCs were West Java (27.6%), West Kalimantan (50.0%), and Papua (54.5%). Meanwhile, the three provinces with the highest availability of SISMALs at PHCs were West Nusa Tenggara (95.5%), South Sulawesi (77.4%), and Central Sulawesi (73.1%).

The availability of the SISMAL differs greatly by DTPKs. PHCs in DTPK areas had a higher level of information system availability than PHCs in non-DTPK areas. Of the 56 PHCs in DTPK areas, 82.1% had available SISMALs. Meanwhile, of the 344 PHCs in non-DTPK areas, only 54.7% had available SISMALs.

The availability of the SISMAL also varies considerably according to regional financial capacity. PHCs with low regional financial capacity had a higher level of information system availability than PHCs with moderate or high regional financial capacity. Of the 201 PHCs with low financial capacity, 62.2% had available SISMALs. Meanwhile, only 54% of the PHCs with moderate or high financial capacity had available SISMALs.

Lastly, the availability of the SISMAL differs significantly according to the endemicity status of the region. PHCs in endemic areas had a higher level of information system availability than PHCs in elimination areas. Of the 109 PHCs in endemic areas, 68.8% had available SISMALs. Meanwhile, of the 291 PHCs in elimination areas, only 54.6% had available SISMALs (Table 1).

Table 1. Availability of Malaria Surveillance Information Systems at Primary Health Centers (n = 400)

Characteristics	Available		Not Available		Total
	N	%	N	%	
**Thematic Province**					
West Java	34	27.6	89	72.4	123
West Kalimantan	14	50.0	14	50.0	28
West Papua	6	54.5	5	45.5	11
Aceh	34	64.2	19	35.8	53
Central Sulawesi	19	73.1	7	26.9	26
South Sulawesi	106	77.4	31	22.6	137
West Nusa Tenggara	21	95.5	1	4.5	22
**Region**					
DTPK	46	82.1	10	17.9	56
Non-DTPK	188	54.7	156	45.3	344

Characteristics	Available		Not Available		Total
	N	%	N	%	
**Regional Financial Capacity**					
Low	125	62.2	76	37.8	201
Moderate	81	54.7	67	45.3	148
High and very high	28	54.9	23	45.1	51
**Endemic Status**					
Elimination	159	54.6	132	45.4	291
Endemic	75	68.8	34	31.2	109
Total	234	58.5	166	41.5	400

3.2. Readiness of Malaria Surveillance Information Systems at Primary Health Centers

SISMAL availability at the PHC does not guarantee readiness in the operational activities of processing and analyzing data as well as presenting and utilizing information related to malaria. For the 234 PHCs (58.5%) with available SISMALs, the level of readiness to run the system only reached 50.2%. Of the eight components of the SISMAL readiness assessment, three components had very low levels of readiness on average: (1) availability of SISMAL personnel (40.9%), (2) SISMAL integration and storage (50.2%), and (3) availability of data sources and indicators (56.8%).

Various problems related to SISMAL personnel at the PHCs included the unavailability of such personnel with the required educational background and the absence of regulations regarding their transfer. Only 9.4% of SISMAL personnel had educational backgrounds in the fields of statistics, epidemiology, computers, or medical records, and the availability of regulations regarding transfers was only 9.8%. Although the PHCs already had a permanent SISMAL staff rate of 78.2%, only 66.2% had received formal training to manage SISMALs.

The main problem related to the unpreparedness of the data sources and indicators is that currently, their level of readiness is only 56.8%. The components that need the top priority for improvement are those that cannot describe the input, process, and output indicators (only 30.8% of the studied PHCs are able to describe them).

Various problems related to SISMAL integration and data storage are that the SISMAL does not use the National Identification Number (NIK) code or the International Classification Code (ICD) code (only 24.6% use NIKs/ICDs), that no bridging exists with other data sources (only 51.7% do data bridging), and that the document retention period is still less than five years (only 34.2% have kept their documents for five years or more). Meanwhile, 91% of PHCs archive their data electronically or in the cloud.

Organizational SISMAL readiness has reached 70.4%. The components that need improvement are organizational structure and job descriptions and functions (readiness of only 45.3%). Only 61.1% of PHCs already have SISMAL organizational structures as well as job descriptions and functions. On the bright side, most PHCs already have special persons in charge of the SISMAL sector (82.9%).

Readiness in the fields of facilities and infrastructure is also low, currently only 62.6%. Of the five components of facilities and infrastructure, three require priority repairs: the availability of standard operational procedures (SOPs) (33.8%), the availability of computers and local arena networks (LANs) (52.6%), and the availability of special funds for the SISMAL (63.2%). The other two components, internet availability (77.4%) and the availability of SISMAL recording standards (85.9%), still have good standing.

Readiness in the fields of data monitoring, evaluation, and verification is very good at 93.2%. Of the 234 PHCs with SISMAL availability, almost 100% (98.7%) have involved

external parties in monitoring and evaluating data, with 97.9% performing data verification activities. NIK, ICD, and CBG verification has reached 90.6%.

Readiness in the field of data quality is also very good at 94.4%. All the data quality components have readiness levels between 93.2% to 95.8%, which include routine, complete, and timely reporting as well as easy-to-retrieve reporting data.

Readiness in the fields of data presentation, utilization, and feedback has reached 90.3%. The component that needs improvement is report feedback (79.5%). The other components still have good standing in terms of readiness, namely, the way to present data (97.9% readiness), the type of media to disseminate information (96.2% readiness), and the use of information by other agencies (89.3% readiness) (Table 2).

Table 2. Readiness of Malaria Surveillance Information Systems at Primary Health Centers (n = 234)

Readiness of SISMAL in Puskesmas	%
**Organization**	**70.4**
- Basic policy of information system: Permenkes + other	92.3
- There is an organizational structure as well as job descriptions and functions	45.3
- There is an organizational structure of the information system	61.1
- There is a person in charge of the information system	82.9
**Human Resources**	**40.9**
- Information systems educational background (statistics/epidemiology/computer/medical records)	9.4
- There is a regulation on the transfer of information systems personnel	9.8
- There is formal training for the person in charge of the information system	66.2
- There is permanent staff for the information system	78.2
**Availability of Infrastructure**	**62.6**
- There are standard operating procedures for presentation and others	33.8
- There are available computers and LANs	52.6
- There is a special fund for the information system	61.5
- There is internet availability	77.4
- There is standard recording of the information system	85.9
**Data Sources and Indicators**	**56.8**
- The information system can describe IPOs and other indicators	30.8
- Data source: Health service + network	82.9
**Monitoring, Evaluation, and Verification**	**93.2**
- MONEV data of information system: Health official + BPJS (moved)	98.7
- There is verification of internal/external data	97.9
- There has been verification of NIKs, ICDs, CBGs, and others	90.6
- There is data verification by the head of the center/director	85.5
**Data Quality**	**94.4**
- There are routine data reports	93.2
- There is complete reporting	94.0
- There is timely reporting	94.9
- The reported data is accurate and easy to obtain	95.8

Readiness of SISMAL in Puskesmas	%
**Presentation, Utilization, and Feedback**	**90.3**
- How to present data (text, tables, graphs, maps)	97.9
- Information media type: Print + online	96.2
- There is report feedback	79.5
- There is the use of information by other institutions	89.6
**Integration and Data Storage**	**50.2**
- The information system already uses the NIK/ICD code	24.6
- Length of data stored: 5+ years	34.3
- Bridging with data: Health official + others	51.7
- Archiving: Electronic/cloud	90.6
**Total Readiness Score**	**70.2**

3.3. Readiness of Malaria Surveillance Information Systems at Primary Health Centers per Province

Out of the seven studied provinces, only two had SISMAL readiness levels above 60%, namely, West Nusa Tenggara and Central Sulawesi. The other five provinces had SISMAL readiness levels of around 50% to 60%, namely, Aceh, West Java, West Kalimantan, South Sulawesi, and Papua.

At the national level, the following components are of particular concern: (1) availability of SIK personnel, (2) availability of data and indicators, and (3) integration of information systems. The foremost problem related to SIK personnel is the unavailability of permanent SIK personnel who are trained and who have educational backgrounds in the fields of statistics, epidemiology, computers, and/or medical records. Meanwhile, the main problem with data and indicators is that the indicators produced have not been able to describe the input, process, and output components. Problems related to information system integration are that the SISMAL does not use NIK and ICD codes, that no bridging exists with other data sources, the data is not filed electronically, and the document storage time is still less than five years.

Apart from the three main issues mentioned above, certain provinces still have problems with the lack of support for the SISMAL's organizational structure, especially in Aceh, West Kalimantan, and South Sulawesi. The provinces that still have difficulty with the use of data and information are West Kalimantan and Papua. Papua also has difficulty with two other areas, namely, infrastructure and verification related to data and information (Table 3).

Table 3. Readiness of Malaria Surveillance Information Systems at Primary Health Centers by Province (n = 234)

Readiness	Aceh n=34	West Java n=34	West Nusa Tenggara n=21	West Kalimantan n=14	Central Sulawesi n=19	South Sulawesi n=106	Papua n=6
K1. Organization	49.3	55.7	65.5	46.4	63.2	44.1	62.5
K2. Human Resources	44.9	42.9	41.7	32.1	51.3	37.3	50.0
K3. Infrastructure	71.8	72.4	75.2	57.1	66.3	54.3	53.3

Readiness	Aceh n=34	West Java n=34	West Nusa Tenggara n=21	West Kalimantan n=14	Central Sulawesi n=19	South Sulawesi n=106	Papua n=6
K4. Indicator	51.0	34.3	33.3	47.6	40.4	34.9	22.2
K5. Verification	56.9	60.8	61.9	52.4	70.2	54.7	47.8
K6. Quality	94.1	90.3	94.0	92.9	84.2	97.9	80.0
K7. Utilization	58.8	64.2	73.8	42.9	63.2	54.0	48.3
K8. Integration	44.9	47.4	45.2	41.1	46.1	48.1	44.2
**K. Total**	**58,9**	**53,2**	**61,3**	**51,6**	**60,6**	**53,2**	**49,8**

3.4. Readiness of Malaria Surveillance Information Systems at Primary Health Centers by Endemicity, DTPK/Remote Area, and Financial Capability

The results of the data analysis showed a normal distribution of SISMAL readiness with a mean value of 55.2% and a median of 53.2% (range 49.8%–61.2%). The multivariate analysis showed that SISMAL readiness at PHCs was significantly related to DTPK area, endemicity status, and regional financial capacity (p-value ≤ 0,001). The SISMAL readiness scores of the districts in remote areas were 4% better than those of districts in non-remote areas. The scores of endemic areas were 1.4% better than those of non-endemic areas. Lastly, the scores of regions with low and moderate financial capacity were better than those of regions with higher regional financial capacity, i.e., 3.78% and 2.91%, respectively (Table 4).

Table 4. Results of ANCOVA Multivariate Analysis on the Readiness of Malaria Surveillance Information Systems at Primary Health Centers

Parameter	Coef.B	Sig	Partial Eta squared
Intercept	50.56	<0.001	0.979
DTPK			
DTPK vs Non-DTPK	4.22	<0.001	0.306
Regional financial capacity			
Low	3.78		
Moderate	2.91	<0.001	0.192
High (ref)		<0.001	0.114
Endemicity			
Endemic vs Elimination	1.4	<0.001	0.073

[1] R-square = 0.493

4. Discussion

Our study shows that the PHCs with the highest scores of information system readiness were in the West Nusa Tenggara province (95.5%), which is committed to achieving malaria elimination by 2019.[15] Hence, the implementation of a malaria surveillance and reporting program is a priority in the province.

The provinces with fairly good availability (60%–80%) were South Sulawesi (77.4%), Central Sulawesi (73.1%), and Aceh (64.2%). This result was supported by a number of studies.[16, 17, 18] A performance report in 2017 on South Sulawesi reported that malaria cases

have been found in majority of districts/cities, i.e., twenty districts/cities with no mortality cases.[16] The malaria incidence rate per 1,000 population was targeted at less than 1. Until the fourth quarter of the reported year, the number of cases was 0.14 per 1,000 population.[16] In 2019, some regions in Central Sulawesi had high endemicity, but other districts achieved malaria elimination, such as Palu, Sigi, Buol, and Toli-toli.[17] Contrarily, in Aceh, only one area was stated as eliminated, while the others had low to high endemicity.[18]

The availability and readiness of the SISMAL were the determinants of the success of the program. We must consider some management elements of the SISMAL as well as the program itself. The support of the central and local governments as well as city districts in controlling malaria with a variety of activities will determine the success of reducing malaria morbidity. Some activities carried out according to the malaria elimination manual are as follows[7]: (1) conducting mass blood surveys (MBSs), (2) conducting epidemiological investigations on each positive case of malaria, (3), carrying out malaria treatments according to standards, (4) conducting malaria vector control surveys,[19] (5) conducting migration surveillance, (6) mapping receptive areas, (7) conducting cross-checks on the results of laboratory examinations, and (8) carrying out efficient recording and reporting. These points are supported by recommendations from Nuraini et al. They stated that the training and practice of E-SISMAL use is to improve the officers' abilities through socialization and that the Lahat Health Office establishes partnerships with telecommunications companies to ensure the smooth running of the network. Developing specific guidelines for the E-SISMAL and a mobile phone–based SISMAL to speed up the malaria reporting process in the Lahat district are also advisable.[26]

The Annual Malaria Research in Indonesia (AMRI) seminar to exchange insights on the future of malaria surveillance, diagnosis, and treatment in the country (held on August 19, 2020) declared the use of online-based applications and digital-based approaches in the malaria program as a form of innovation. The Province Health Office (PHO) of East Nusa Tenggara presented the use of an electronic and online malaria readiness self-assessment tool as relevant to malaria elimination efforts. Using this tool, health workers could track the progress of malaria intervention activities from the PHC up to the province level, with the aim to achieve a malaria-free district.[5]

The role of the local government is important as well.[19] In Central Sulawesi Province, the government enacts policies for malaria elimination, as stated in the Governor's Decree No. 20/2012[17] (Decree of the Minister of Health of the Republic of Indonesia No. 293/MENKES/SK/IV/2009, dated April 28, 2009, regarding the elimination of malaria in Indonesia, and the Minister of Home Affairs Circular No. 443.41/465/SJ of 2010 regarding the implementation of the malaria elimination program in Indonesia[5, 9, 13]). The requirements to obtain a malaria elimination certificate are as follows: (1) no cases of indigenous (local) transmission for three consecutive years, (2) an annual parasite incident (API) rate of <1‰ for three consecutive years, and (3) a slide positive rate (SPR) of <5% for three consecutive years.[5, 9, 13]

The readiness of SISMALs in PHCs in major areas was below the national average (below 70.2%). Meanwhile, some areas achieved readiness rates approaching 60%, including West Nusa Tenggara, Central Sulawesi, and Aceh. The problems observed were human resources, data storage components and integration, and SISMAL indicators.

The issues related to human resources were education and human resource resistance. These findings were in accordance with the results of Darman.[8, 21, 22, 23, 24, 25, 27, 28] However, the issues related to data integration and storage components were caused by the suboptimal use of the civil ID registration number, which worsened interoperability. These findings were in accordance with policy studies on the governance of health information system resources in the implementation of PMK No. 97 of 2015.[21] One study shows that interoperability not only helps improve patient safety and quality of care but also reduces the time and resources spent on data format conversion between hospitals. Other studies focus on the importance of an integrated SISMAL.[22, 29, 30, 32, 33, 34] Even the existence

of an information system equipped with GIS is highly recommended for various infectious diseases such as malaria.[31, 35, 38]

The problems with regard to data sources and indicators were the indicators of the input and output processes of the program, as supported by previous studies.[22, 30] In one paper, this indicator is termed a "data warehouse."[35, 36] The vivax working group meeting routine report states that several general data indicators are needed in controlling malaria.[38] We attempted to address this problem and found that the data and information related to environmental factors such as rainfall rate, vector data, etc. were excluded and could not be reported as input data.

Remote area and regions with low financial capacity were more accessible in terms of readiness and availability. Endemic areas had more SISMAL availability than eliminated areas. Malaria could occur in many DTPK areas that lack financial capacity. Thus, the SISMAL is needed for the implementation of the program.

5. Conclusions

The SISMAL is available at only 58.5% of PHCs. Of this percentage, only 56.0% are ready to run it well. SISMAL readiness at the PHCs was significantly related to DTPK area, endemicity status, and regional financial capacity. DTPK areas, endemic areas, and regions with low or medium financial capacity have better levels of SISMAL readiness than non-DTPK areas, areas of elimination, and regions with high financial capacity.[32] Some studies have demonstrated that SISMAL feasibility could significantly improve access to low-cost, valuable, and safe diagnoses.[37] Traditional epidemiological surveillance in other studies and the approach presented in this paper are complementary, but a formal validation framework for case identification algorithms is necessary.

6. Patents

This section is not mandatory but may be added if there are patents resulting from the work reported in this manuscript.

Supplementary Materials: The following supporting information can be downloaded at: www.mdpi.com/xxx/s1, Figure S1: title; Table S1: title; Video S1: title.

Author Contributions: Conceptualization, M.H.H.; methodology, M.H.H. and B.; software, B.; validation, B. and A.A.; formal analysis, B.; investigation, M.H.H. and M.V.; resources, M.H.H.; data curation, M.H.H., A.A., and M.V.; writing—original draft preparation, M.H.H. and M.V.; writing—review and editing, B. and A.A.; visualization, B.; supervision, M.H.H., A.A. and M.V.; project administration, M.H.H.; funding acquisition, M.H.H. All authors have read and agreed to the published version of the manuscript.

Funding: This research was funded by NIHRD Ministry of Health Republic of Indonesia.

Institutional Review Board Statement: The study was conducted in accordance with the Declaration of Helsinki, and approved by the Institutional Review Board (or Ethics Committee) of (Protocol Code: Leader of Centre of Research and Development for Human Resources and Health Services, National Institute for Health Research and Development, Indonesian Ministry of Health, No. HK.03.02/1/180/2019, and Ethical Approval LB.02.01/2/KE.186/2019).

Informed Consent Statement: Informed consent was obtained from all subjects involved in the study.

Data Availability Statement: The details data in this study can be found publicly in Data Management Laboratory of NIHRD – Ministry of Health.

Acknowledgments: Our gratitude to all field technical person in charge as well as field enumerators that were contributing in data collection. We also thank to all provincial and regional government including health offices in the respective area of research.

Conflicts of Interest: The authors declare no conflict of interest.

Appendix A

The appendix is an optional section that can contain details and data supplemental to the main text—for example, explanations of experimental details that would disrupt the flow of the main text but nonetheless remain crucial to understanding and reproducing the research shown; figures of replicates for experiments of which representative data is shown in the main text can be added here if brief, or as Supplementary data. Mathematical proofs of results not central to the paper can be added as an appendix.

Appendix B

All appendix sections must be cited in the main text. In the appendices, Figures, Tables, etc. should be labeled starting with "A"—e.g., Figure A1, Figure A2, etc.

References

1. Farrar J. Hotez Peter J, Junghanss T, Kang G, Lalloo, David, White Nicholas J, Manson's, Tropical Diseases Twenty- Third Edition. Elsevier Saunders. 2014. 1-1360.
2. Center for Multilateral Policy Assessment and Development Agency for Policy Assessment and Development of the Ministry of Foreign Affairs of the Republic of Indonesia. Independent Study, "Health for All: Indonesia's Global Health Diplomacy Strategy" Jakarta. 2018. downloaded on 7 August 2021 at 13:42 PM from https://kemlu.go.id/download/L3NpdGVzL3B1c2F0L0RvY3VtZW50cy9LYWppYW4lMjBCUFBLL1AzSyUyME9JLU1VTFRJTEFURVJBTC8wNl9LZXNlaGF0YW5fUGRmVtWdWEfUGRmVtWtFUc2W50.
3. Budiman R. History of Malaria Handling Since Colonial-Orba, Kompasiana beyond Bogging 2015. downloaded on 19 October 2020 at 13:45 PM from https://www.kompasiana.com/ryakair/5516d9368133112d52bc6d8c/sejarah-penanganan-malaria-sejak-kolonial-orba?page= all.
4. Pratiwi, Puput Akad Ningtyas. History of National Health Day Every November 12. Starting with President Soekarno's Efforts to Eradicate Malaria. 2018. downloaded on October 19, 2020, at 13:45 PM, from https://www.grid.id/read/04996394/sejarah - national-health-day-every-12-november-starts-from-presidential-soekarno-eradicating-malaria?page=3.
5. Ministry of Health. Republic of Indonesia. Factsheet on the Current Malaria Situation in 2020. Report on the Latest Situation on the Development of the Malaria Control Program in Indonesia in 2019. Downloaded on October 19, 2020, at 1:45 p.m. from http://www.malaria.id/p/blog-page_43. html.
6. WHO. Tool Kit Monitoring Health Systems Strengthening, Health Information System, June. 2008. 1-9.
7. Republic of Indonesia. Law of the Republic of Indonesia Number 4 of 1984. On Outbreaks of Infectious Diseases. Jakarta. 1984.
8. Zayadan D. Malaria Information System Development at the Health Office of South Lampung City, Lampung Province in 2009. Thesis. Public Health Study Program. FKM UI. Depok. West Java. Indonesia. July. 2009. 1-108.
9. Republic of Indonesia, KMK. RI number 293/Menkes/SK/IV/2009 concerning Elimination Malaria in Indonesia. Jakarta. Indonesia. 2009.
10. Sariwati E. In Power Point. Malaria Surveillance Policy. Malaria Sub-Directorate. In the Socialization of the Malaria Surveillance Information System Version 2. Bekasi. 26 February 2018.
11. **AMRI**, What Will The Future Look Like For Malaria Surveillance, Diagnosis And Treatment?. 26 April 2021 downloaded on 10 August 2021. 21.03 PM. https://www.who.int/indonesia/news/detail/26-04-2021-what-will-the-future-look-like-for-malaria-surveillance-diagnosis-and-treatment,
12. Riau. Politics and Governance. The Importance of Electronic Malaria surveillance Information System E-SISMAL for Malaria Program Processing. 2015. Downloaded on October 20, 2020, at 11.06 am from https://www.riau.go.id/home/skpd/2016/08/12/1823-penting-elektronik-sistem-information-surveilans-malaria-e-sismal-bagi - processing
13. Ministry of Health, Infodatin Indonesian health data and information center ISSN 2442-7669, Malaria, 10 April, World Malaria Day@Pusdatin. Jakarta. Indonesia. 2016
14. Herawati M.H. et al. Research Report. Thematic Rifaskes, "Availability and Readiness of Health Information Systems in Health Services in the Implementation of National Health Insurance in One Data". Balitbangkes. Ministry of Health. Jakarta. 2019.
15. Datin Research and Development. Towards NTB Malaria Elimination. 2018.Downloaded from https://dinkes.ntbprov.go.id/article/menuju-ntb-elimination-malaria/. 15-04 2021 at 15.18
16. South Sulawesi Health Office. Health Profile of South Sulawesi Province in 2018.2018
17. Central Sulawesi Health Office.Health Profile of Central Sulawesi Province in 2018.2019
18. Zohra. Aja Fatimah, Anwar, Samsul, Fitri. Aida, Nasution. Muhammad Haikal. Regional Classification of Aceh Province Based on Vulnerability Levels of Malaria Cases in 2015 – 2018.JKLI. Indonesian Journal of Environmental Health. 18(1), 2019, 25 – 33.
19. Rodriguez, Julio Cesar Padila. Olivera, Mario J. Franco, Martha Liliana Ahumada. Medina.Andrea Elizabeth Paredes. Malaria risk stratification in Colombia 2010 to 2019. PLOS ONE | https://doi.org/10.1371/journal.pone.0247811 March 11, 2021.
20. Regional Regulation, Ambon City. Maluku Province. No. 1 of 2018 concerning the Elimination of Malaria. Ambon, May 3, 2018

21. Herawati, Maria Holly, et al., Policy Review Report "The Governance of Health Information System Resources in the Implementation of PMK no. 97 of 2015", Balitbangkes. Ministry of Health. Jakarta. 2020.
22. Zainuddin and Lucia Yovita Hendrati. Evaluation of the Implementation of the Malaria Surveillance System at the Sumbawa Besar District Health Office. Periodic Journal of Epidemiology, Vol. 2, No. September 3, 2014: 342–354.
23. Astuti, Endang P., Ipa, Mara., Ginanjar, Aryo., Wahono, Tri,. Malaria Control Efforts for Pre-Elimination in Garut Regency: A Qualitative Study. Malaria Control Efforts On Pre-Elimination In Garut Regency: A Qualitative Study. Health Systems Research Bulletin – Vol. 22 No. October 4, 2019: 255–264.
24. Capah, Tohap., Study on Environmental Management Planning in Malaria Control Program in Asmat District, 2008, Thesis, Postgraduate Program at Diponegoro University, Semarang, 2008, 170 pages.
25. Aqsa, Silvia Nurul. Process of Management of Malaria Control Program at PHC West Sei Kepayang, Asahan Regency. Thesis. Faculty of Public Health. University of North Sumatra. Medan 2016, 92 pages.
26. Nuraini,Eni. Hasyim, Hamzah.Idris, Haerawati, Ghiffari, Ahmad.Anwar, Chairil.Analysis of The Electronic Implementation of A MalariaSurveillance Information System (E-SISMAL)in the Lahat District. Bioscientia Medicine: Journal of Biomedicine and Translational Research: eISSN (Online): 2598-0580 . Journal homepage: www.bloscmed.com Page 1078-1084 diakses Kamis 16 Desember 2021 jam 21:48 dihttps://www.bioscmed.com/index.php/bsm/article/view/419/578
27. Randriamiarana, Rado. Raminosoa, G.Vonjitsara, N. Randrianasolo, Rivo.Rasamoelina, Harena, Razafimandimby,H. Rakontonjanabelo, A.L.Lepec, R. Flachet, L.Halm, A. BMC Health Service Research(2018) 18:265. https://doi. Org/10.1186/s12913-018-3081-2
28. Larocca, A. Visconti, RM. Marconi, M. Malaria Diagnosis and Mapping With m-Health and Geographic Information Systems (GIS): Evidence From Uganda. Malaria Journal (2016) 15:520 DOI.10.1186/s12936-016-1546-5
29. Rosewell, Alexander. Makta,Leo. Muscatello, David. John, Lucy Ninmongo. Bieb, Sibauk. Hutton, Ross. Ramamurthy, Sundar. And Shearman, PhilHealth Information System Strengthening and Malaria Elimination in Papua New Guinea. Malaria Journal *(2017) 16:278* DOI 10.1186/s12936-017-1910-0
30. Ohrt, Colin. Roberts, Kathryn W. Sturrock, Hugh J.W. Wegbreit, Jennifer. Lee, Bruce Y.Lee. Gosling, Roly D. Information System to Support Surveillance for Malaria Elimination. Amrican Journal. Tropical Medicine. Hyg, 93 (1), 2015, pp 145-152. Doi: 10.4269/ajtmh.14-0257
31. Kim, Jung-Yoon. Eun, Sung-Jong and Park, Dong Kyun. Malaria Vulnerability Map Mobile System Development GIS-Based Decision-Making Technique.Hindawi Mobile Information Systems. Volume 2018. Article ID 8436210. 9 pages. https://doi.org/10.1155/2018/8436210.
32. Asmiani. Windusari, Yuanita. Hasyim, Hamzah. Malaria Vector Control and The Electronic Malaria Surveillance Information System (E-Sismal) in Bangka Barat Regency Indonesia. Journal Kesehatan Lingkungan vol 13. No. 4DOI: 10.20473/jkl.v13i4.2021.241-249. ISSN:1829-7285 E-ISSN:2040-881X.
33. Tiffany, Amanda.Moundekeno,Faya Pascal.Traore, Alexis. Haile, Melat.Sterk, Esther. Guilavogui, Timothe. Serafini, Micaela.Genton, Blaise.Grais, Rebecca F. Community –Based Surveillance to Monitor Mortality in a Malaria-Endemic and Ebola-Epidemic Setting in Rural Guinea. Amirican Journal Tropical Medicine and Hygiene. 95 (6), 2016, pp.1389-1397. Doi:10..4269/ajtmh.16-0376.
34. Streatfield, P.Kim etc. Malaria Mortality in Africa and Asia: Evidence from INDEPTH Health and Demographic Surveillance System Sites. Global Health Action. 2014, 7, 25369 – http://dx.doi.org/10.3402/gha.v7.25369. INDEPTH Network Cause-Specific Mortality
35. Ichsan. Satoto, Tribaskoro Tunggul. Widartono, Barandi Sapta. Overview of Physical Environmental Factors in Malaria Vulnerability Areas in Boul Regency. Journal of Information Systems for Publich Health, Vol 2, No. 2, Agustus 2017. Halaman 48-54.
36. Kardiana, Aan. Diana, Nova Eka. Visualization of Ontology-Based Data Warehouse For MalariaSpread Incidences UsingProtege.Journal Sistem Informatsi (journal of Information Systems). 1/12 (2016), 9-14. DOI: http://dx.doi.org/10.21609/jsiv12iI.452.
37. Hutapea, MMRS. Simanjorang, R.M. Expert System to Diagnose Malaria With Bayes Method. Journal of Computer Networks, Architeccture and high performance computing. E-ISSN 2655-9102. Volume 2, No. 1 January 2020. Pp 88-92.
38. Booman, Marlize.Durrheim, Dave N.Grange, Kobus La.Martin, Carrin. Mabuzza, Aaron M.Zitha, Alpheus. Mbokazi, Frans M.Fraser, Collen, Sharp, Brian L.Using a Geographical InformationSystem to a Malaria Control Programme in South Africa. Bulletin of the World Health Organization, 2000, 78: 1438–1444.
39. Ruwanpura, VSH. Nowak,Spike. Guyette, EG. Theodora,M. Dysoley, L. Haile, M.Grieetens, KP. Price, RN. Lynch, CA. Thriemer, K. Further Evidence Needed To Change Policy For The Safe and Effective Radical Cure of Vivax Malaria: Insights From The 2019 annual APMEN Vivax Working Group Meeting. Asia dan the Pasific Policy Studies, 2021;8;208-242. Published by Crawford School of PublicPolicy of the Australian National University and John Wiley and Sons Australia. Ltd.
