# Supplementary material for: Service availability and readiness of malaria surveillance information systems implementation at primary health centers in Indonesia
Source: PLoS One. 2023 Apr 27;18(4):e0284162. doi: 10.1371/journal.pone.0284162 (PMC10138467; doi:10.1371/journal.pone.0284162)
Supplement: Attachment — Submitted filename: 2nd Letter of Revision SISMAL PLOS ONE.docx [file pone.0284162.s003.docx]

**2^nd^ Letter of Revision**

PONE-D-22-07674

Service Availability and Readiness of Malaria Surveillance Information Systems Implementation at Primary Health Centers in Indonesia

PLOS ONE

>>Dear Dr. Luzia Helena Carvalho, Ph.D. - Academic Editor of PLOS ONE,

Thank you very much for your continuous support in our research’s manuscript. Our gratitude also to the Reviewers that have been made substantial and constructive reviews and suggestions to our manuscript.

Please find below our rebuttal point-by-point revision according to the Reviewers-minor revisions’ comments.

Best wishes,

Meita Veruswati – Corresponding author

Dear Dr. Veruswati,

Thank you for submitting your manuscript for review to PLoS ONE. After careful consideration, we feel that your manuscript will likely be suitable for publication if the authors revise it to address critical points raised now by the reviewer.  According to reviewer, there are some specific areas where further improvements would be of substantial benefit to the readers.A copy of the reviewers’ comments was included for your information.  

Please submit your revised manuscript by February 20. If you will need more time than this to complete your revisions, please reply to this message or contact the journal office at [plosone@plos.org](mailto:plosone@plos.org). When you're ready to submit your revision, log on to https://www.editorialmanager.com/pone/ and select the 'Submissions Needing Revision' folder to locate your manuscript file.

Please include the following items when submitting your revised manuscript:

- A rebuttal letter that responds to each point raised by the academic editor and reviewer(s). You should upload this letter as a separate file labeled 'Response to Reviewers'.
- A marked-up copy of your manuscript that highlights changes made to the original version. You should upload this as a separate file labeled 'Revised Manuscript with Track Changes'.
- An unmarked version of your revised paper without tracked changes. You should upload this as a separate file labeled 'Manuscript'.

If you would like to make changes to your financial disclosure, please include your updated statement in your cover letter. Guidelines for resubmitting your figure files are available below the reviewer comments at the end of this letter.

If applicable, we recommend that you deposit your laboratory protocols in protocols.io to enhance the reproducibility of your results. Protocols.io assigns your protocol its own identifier (DOI) so that it can be cited independently in the future. For instructions see: <https://journals.plos.org/plosone/s/submission-guidelines#loc-laboratory-protocols>. Additionally, PLOS ONE offers an option for publishing peer-reviewed Lab Protocol articles, which describe protocols hosted on protocols.io. Read more information on sharing protocols at <https://plos.org/protocols?utm_medium=editorial-email&utm_source=authorletters&utm_campaign=protocols>.

We look forward to receiving your revised manuscript.

Kind regards,

Luzia H Carvalho, Ph.D.
Academic Editor
PLOS ONE

Journal Requirements:

Please review your reference list to ensure that it is complete and correct. If you have cited papers that have been retracted, please include the rationale for doing so in the manuscript text, or remove these references and replace them with relevant current references. Any changes to the reference list should be mentioned in the rebuttal letter that accompanies your revised manuscript. If you need to cite a retracted article, indicate the article’s retracted status in the References list and also include a citation and full reference for the retraction notice.

[Note: HTML markup is below. Please do not edit.]

Reviewers' comments:

Reviewer's Responses to Questions

**Comments to the Author**

1. If the authors have adequately addressed your comments raised in a previous round of review and you feel that this manuscript is now acceptable for publication, you may indicate that here to bypass the “Comments to the Author” section, enter your conflict of interest statement in the “Confidential to Editor” section, and submit your "Accept" recommendation.

Reviewer #3: (No Response)

Reviewer #4: All comments have been addressed

2. Is the manuscript technically sound, and do the data support the conclusions?

The manuscript must describe a technically sound piece of scientific research with data that supports the conclusions. Experiments must have been conducted rigorously, with appropriate controls, replication, and sample sizes. The conclusions must be drawn appropriately based on the data presented.

Reviewer #3: Yes

Reviewer #4: Yes

3. Has the statistical analysis been performed appropriately and rigorously?

Reviewer #3: Yes

Reviewer #4: Yes

4. Have the authors made all data underlying the findings in their manuscript fully available?

The [PLOS Data policy](http://www.plosone.org/static/policies.action#sharing) requires authors to make all data underlying the findings described in their manuscript fully available without restriction, with rare exception (please refer to the Data Availability Statement in the manuscript PDF file). The data should be provided as part of the manuscript or its supporting information, or deposited to a public repository. For example, in addition to summary statistics, the data points behind means, medians and variance measures should be available. If there are restrictions on publicly sharing data—e.g. participant privacy or use of data from a third party—those must be specified.

Reviewer #3: Yes

Reviewer #4: Yes

5. Is the manuscript presented in an intelligible fashion and written in standard English?

PLOS ONE does not copyedit accepted manuscripts, so the language in submitted articles must be clear, correct, and unambiguous. Any typographical or grammatical errors should be corrected at revision, so please note any specific errors here.

Reviewer #3: Yes

Reviewer #4: Yes

6. Review Comments to the Author

Please use the space provided to explain your answers to the questions above. You may also include additional comments for the author, including concerns about dual publication, research ethics, or publication ethics. (Please upload your review as an attachment if it exceeds 20,000 characters)

**Reviewer #3:**

The manuscript presented by Meita Veruswati et al. reports on Service Availability and Readiness of Malaria Surveillance Information Systems Implementation at Primary Health Centers in Indonesia. The authors present a descriptive assessment of the availability and readiness of the electronic malaria surveillance information system (E-SISMAL).

In general, the manuscript is well written, interesting, and very useful to the malaria community. I have only few issues which need addressing/clarification.

>>Dear Reviewer #3: Dr. Joseph J. Joseph,

Thank you very much for your appreciation to our manuscript. Our gratitude also for your substantial and constructive reviews and suggestions to our manuscript.

Please find below our rebuttal point-by-point revision according to your-minor revisions’ comments below.

Minor issues

1. The author should briefly describe in method, what malaria data is being collected by E-SISMAL system and the frequency of collection, is it daily, weekly or monthly? Does it collecting routine malaria data, case-based surveillance system (CBS) or non-routine? Is it the only system for malaria surveillance in Indonesia? If yes, how does malaria data being collected in the areas where E-SISMAL/SISMAL is not implemented?
This is probably beyond the scope of this manuscript, but at least to understand the process of malaria surveillance in Indonesia.

>> Thank you for your comment. We have already provided these information in the introduction section particularly in the paragraph 4, in order to understand the process of malaria surveillance in Indonesia. And, yes, we agree with you that add information about its frequency collection (i.e. monthly reported to E-SISMAL) (line 78), with routine CBS. Currently, E-SISMAL/SISMAL is only implementing in Indonesia, however, we have already discussed about other malaria information system with CBS in other countries such as in low-resource setting countries. Thank you.

2. In line 25: I like the way the authors present the result by strata, but the authors should clarify how these strata (low, high, and moderate) financial capacity is defined. The definition can be included here or in the method.

>> Thank you for your suggestion. We have now clarified these strata of district financial capacity index (IKFD) that set by Indonesian Ministry of Finance in the method section (line 114-115).

3. Line 76; Author mentioned application implemented in several districts, please indicate the number of districts in which SISMAL was implemented. And how many districts were targeted. To understand the coverage for E-SISMAL implementation at that time (2012).

>> Thank you. We have now added the regions that implemented as the first of four-phase of 2030 elimination program in Indonesia.

4. In Line 270; I advise author to include this reference from WHO
"WHO Malaria elimination framework 2017" https://apps.who.int/iris/bitstream/handle/10665/254761/9789241511988-eng.pdf

>> Done! Thank you.

5. In discussion section: The author explained very well the result of SISMAL, however I feel that the discussion could be strengthened a bit i.e., To describe how the NMCP /government can improve the availability of SISMAL in across all PHC. example the SISMAL now is implemented as standalone/web app which operate within the specific facility. There is need for a government to find a best approach to role out the system to all health facilities and have standardize and centralized system for malaria surveillance across the country.

>> Thank you. The essential effort of the government to establish SISMAL in all health facilities has been elaborate in the discussioin section particularly started in line 309. We acknowledge the need of potential improvement for this system including the utilization of GIS (line 330) as well as mobile phone-based (line 292). And yes, we agree with you about standardize the system through data source/input parameters and indicators (line 332) that we have identified as the problem of this surveillance information system in varies and huge of Indonesia’s setting.

**Reviewer #4:**

In this manuscript, Herawati et al. present the results of a descriptive study of a Malaria Epidemiological Information System (SISMAL) in Indonesia. Basically, the authors describe the proportions of information´s availability and readiness in 400 health units randomly chosen among those that provide primary health care in 7 provinces spread across the country.
I revised a version of the manuscript in which I found a tracked changes revision performed by reviewer 1, along with all response from the authors were showed to all his comments. All the reviewer´s comments were addressed by the authors.
Although it is an essentially descriptive study, the authors carried out a comparative analysis to assess the association between the proportions of availability and readiness of the information system (SISMAL) with some characteristics related to the provinces where the health units are located, such as location (remote and border área), regional financial capacity, and regional status according to the malaria program (under elimination or
The manuscript is well written, with correct and clear English. The study methodology is adequate to respond to the study objectives. The data are well analyzed and the conclusion of the study is adequate to the observed results.

>>Dear Reviewer #4,

Thank you very much for your appreciation to our manuscript.

The only point that I consider necessary to clarify in the Abstract is the authors' statement that "the readiness of SISMAL in these UBS is significantly related to the DTPK area, endemicity status and financial capacity”. In this sentence, it is important to clarify which categories of these variables were, in fact, associated with SISMAL readiness. That is, what were the categories of variables that were associated? Central or remote location? Endemic or elimination area? High or low financial capacity?

>> Thank you for your comments. We have now provide its classification that is associated with the readiness of SISMAL into: “the readiness of SISMAL in these UBS is significantly related to the DTPK/remote area, high endemicity status and low financial capacity”. These classification are defined in method sections.

7. PLOS authors have the option to publish the peer review history of their article ([what does this mean?](https://journals.plos.org/plosone/s/editorial-and-peer-review-process#loc-peer-review-history)). If published, this will include your full peer review and any attached files.


If you choose “no”, your identity will remain anonymous but your review may still be made public.


**Do you want your identity to be public for this peer review?** For information about this choice, including consent withdrawal, please see our [Privacy Policy](https://www.plos.org/privacy-policy).

Reviewer #3: **Yes:** Joseph J. Joseph

Reviewer #4: No
